# Subcortical volume in middle-aged adults with fetal alcohol spectrum disorders

**DOI:** 10.1093/braincomms/fcae273

**Published:** 2024-09-03

**Authors:** Amanda Bischoff-Grethe, Susan A Stoner, Edward P Riley, Eileen M Moore

**Affiliations:** Department of Psychiatry, University of California, San Diego, La Jolla, CA, 92093, USA; Department of Psychiatry and Behavioral Sciences, Fetal Alcohol and Drug Unit, University of Washington School of Medicine, Seattle, Washington 98105, USA; Department of Psychology, Center for Behavioral Teratology, San Diego State University, San Diego, CA, 92120, USA; Department of Psychology, Center for Behavioral Teratology, San Diego State University, San Diego, CA, 92120, USA

**Keywords:** FASD, prenatal alcohol, neuroimaging, aging

## Abstract

Studies of youth and young adults with prenatal alcohol exposure (PAE) have most consistently reported reduced volumes of the corpus callosum, cerebellum and subcortical structures. However, it is unknown whether this continues into middle adulthood or if individuals with PAE may experience premature volumetric decline with aging. Forty-eight individuals with fetal alcohol spectrum disorders (FASD) and 28 healthy comparison participants aged 30 to 65 participated in a 3T MRI session that resulted in usable T_1_-weighted and T_2_-weighted structural images. Primary analyses included volumetric measurements of the caudate, putamen, pallidum, cerebellum and corpus callosum using FreeSurfer software. Analyses were conducted examining both raw volumetric measurements and subcortical volumes adjusted for overall intracranial volume (ICV). Models tested for main effects of age, sex and group, as well as interactions of group with age and group with sex. We found the main effects for group; all regions were significantly smaller in participants with FASD for models using raw volumes (*P*’s < 0.001) as well as for models using volumes adjusted for ICV (*P*’s < 0.046). Although there were no significant interactions of group with age, females with FASD had smaller corpus callosum volumes relative to both healthy comparison females and males with FASD (*P*’s < 0.001). As seen in children and adolescents, adults aged 30 to 65 with FASD showed reduced volumes of subcortical structures relative to healthy comparison adults, suggesting persistent impact of PAE. Moreover, the observed volumetric reduction of the corpus callosum in females with FASD could suggest more rapid degeneration, which may have implications for cognition as these individuals continue to age.

## Teaser

Bischoff-Grethe *et al*. examined subcortical volumes in middle-aged adults with fetal alcohol spectrum disorders and reported reduced volumes in examined regions relative to healthy comparison adults, suggesting a persistent impact of prenatal alcohol exposure.

## Graphical Abstract

**Graphical Abstract:**
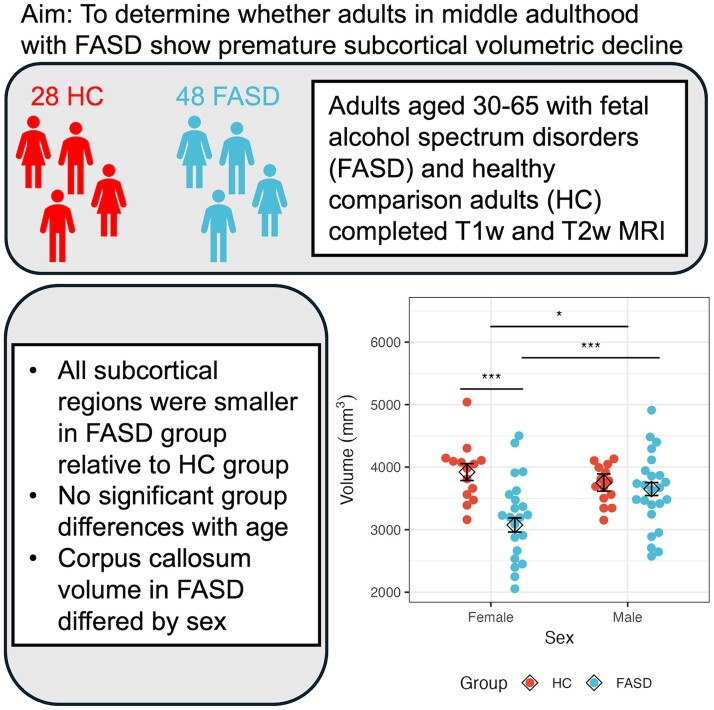

## Introduction

Fetal alcohol spectrum disorders (FASD) refer to a range of adverse outcomes caused by prenatal alcohol exposure (PAE). While these outcomes can include altered physical features, including growth restriction, major malformations that primarily affect cardiac and ocular functioning, and facial dysmorphism, functional consequences of PAE’s effect on the brain often cause major difficulty for affected individuals.^1^ Research has found that the brain is especially sensitive to the adverse effects of alcohol during prenatal development, and the vast majority of individuals affected by PAE experience some degree of cognitive, behavioural and/or neurological deficit.^2^ The timing, duration and dose of alcohol during gestation, as well as nutrition, stress and genetics, among other factors, interactively influence both prenatal and postnatal brain development. As a result, FASD is characterized by a wide range of adverse outcomes.^3^ It is estimated that PAE affects roughly 1–5% of children within the United States, 0.7% globally and 10% or more in some countries.^4-6^

Brain structure has been well studied in children and adolescents with FASD. While results can differ depending upon age and other characteristics, several findings have been fairly consistent across studies. Heavy PAE is associated with generally smaller brain volumes; this effect is particularly pronounced in children with fetal alcohol syndrome (FAS),^7^ which is diagnosed based on the presence of cardinal facial features, growth restriction and evidence of central nervous system (CNS) disruption.^8^ Above and beyond what would be expected in an overall smaller brain, regional effects have also been reported. In particular, disproportionate effects have commonly been observed in the corpus callosum, cerebellum and basal ganglia of children and adolescents with FASD.^9^ A few studies have examined young adults (reviewed in^10^). Chen *et al*.^11^ performed a whole-brain analysis and found that the bilateral caudate and lingual gyri as well as the left cuneus cortex and right inferior temporal gyrus were disproportionately reduced in young adults with FAS as compared with controls. Additionally, a series of studies conducted region of interest (ROI) analyses in a sample of adolescents and young adults with FASD.^12-15^ Volumetric analysis found that the corpus callosum, cerebellum, caudate and putamen were disproportionately smaller in those with FASD vs. controls.^15^ Shape analysis of the corpus callosum suggested greater shape variability in the FASD group,^12,13^ but a separate analysis of the cerebellum did not find shape variability between the HC and FASD groups.^16^ Within the cerebellum, disproportionate effects were observed in VIIB and VIIIA for those with FASD regardless of whether they met the criteria for FAS. For those who met criteria for FAS, lobules I, II, IV, V and VI and Crus II were additionally affected.^14^ Inkelis *et al*.^15^ examined the relationship between age and volume in individuals aged 13 to 30 years and found evidence that the caudate, corpus callosum and cerebellum may have altered volumetric trajectories for those with FASD. In the caudate and corpus callosum, an inverted U-shape relationship between age and volume was noted such that group differences were more robust among the youngest adolescents and the oldest young adults. In the cerebellum, a linear decline in volume with age was noted in the FASD group but not in controls. While this was a cross-sectional study, it led to a concern that accelerated brain aging may occur in persons with FASD and a realization that older age ranges needed to be evaluated.

Brain maturation is a complex phenomenon, and while there are generalizable features, it occurs in a nonlinear fashion,^17-19^ is spatially distributable and varies among individuals.^20-22^ In healthy children, the rate of development in motivational (ventral) circuits within the striatum peaks in mid- to late adolescence, whereas higher-order cognitive control (dorsal) cortical circuits peak in late adolescence/young adulthood.^23,24^ Similar to the cortex, the corpus callosum and cerebellum both undergo rapid development in early childhood, slower growth during adolescence and early adulthood, a period of stability in middle adulthood, and volumetric decline in later adulthood.^25,26^ This trajectory may differ in individuals with FASD, as evidenced by their elevated levels of impulsivity and emotion dysregulation.^27^ Although the literature suggests atypical brain maturation in children with FASD, the interpretation is mixed. Some longitudinal studies of children and adolescents found that both children with PAE and healthy controls showed similar volumetric increases between two neuroimaging visits at least two years apart,^28^ while others found that healthy controls, but not children with FASD, showed age-related increases in total white matter as well as in the pallidum and amygdala.^29^ In contrast, cross-sectional studies suggest that there may be atypical development during distinct childhood periods and within subcortical regions.^30^ For example, both young and early adolescent children with PAE had smaller caudate and putamen volumes relative to typically developing children, whereas older adolescents with PAE were not significantly different from typical adolescents. As noted earlier, cross-sectional work that included both adolescents and young adults found differential effects of age on subcortical volumes in individuals with FASD.^15^ For individuals with FASD, older age was associated with smaller total volume within the corpus callosum, caudate and cerebellum, but this relationship was not seen in the control group. However, interpretability of these findings is limited, as at least three time points are required to assess trajectories of neurological change.^31,32^ Thus, while children and young adults with FASD may potentially show altered subcortical volumes and neurodevelopmental trajectories, it is unclear whether these changes extend into or are exacerbated in older adulthood.

Another apparent difference in brain morphology is a lack of sexual dimorphism observed in children and adolescents with FASD, including for subcortical structures such as the caudate, putamen and pallidum.^15,33,34^ This is in contrast to studies of typically developing youth, which report diverging growth trajectories and increases in sexual dimorphism during adolescence into early adulthood.^35-37^ Typically developing males tend to have larger volumes for the pallidum, putamen and cerebellum, whereas females appear to have larger volumes for the caudate,^38,39^ even after controlling for overall differences in total brain volume. Some investigators have suggested the corpus callosum may also exhibit sexual dimorphism in healthy populations,^40^ but others have disputed this finding, instead suggesting these differences are more likely due to differences in brain size rather than sex.^41,42^ It is currently unknown whether attenuated sex differences persist into older adulthood in individuals with FASD.

To date, few neuroimaging studies of FASD have included adult subjects, and they have generally focused on young adults. Neurobehavioural studies have reported cognitive impairment in children with FASD relative to children without FASD,^2^ and recent work suggests these impairments may continue to be present in adults with FASD.^43^ However, it is unknown whether the altered developmental trajectory observed in children and adolescents continues throughout the lifespan. We therefore performed a cross-sectional analysis in adults aged 30 to 65 with and without FASD, 88% of whom had participated in prior studies.^13,15^ Our goals were to (i) determine whether adults with FASD continue to show reduced brain volumes relative to adults without FASD, (ii) assess whether age interacts with FASD status in affecting brain volumes, and (iii) examine whether sex differences in brain volumes are attenuated in older adults with FASD. We hypothesized that, compared with controls, adults with FASD would exhibit smaller brain volumes, both overall and regionally; earlier age-related volumetric decline; and attenuated sexual dimorphism. If volumetric reduction persists or accelerates in middle-aged adults with FASD, it may have profound implications for the lifespan trajectory of overall brain health and cognitive functioning, including the potential for future complications as individuals with FASD continue to age.

## Materials and methods

### Participants

Fifty-six adults with FASD and thirty-one healthy comparison adults (HC) who were part of a larger study examining the effects of PAE on middle-aged adults^44^ were recruited to participate in a neuroimaging sub-study. A majority of participants (51 FASD, 26 HC) had also participated in a prior study, called the FASD Follow-up Study, which began over 40 years ago at the University of Washington and included a structural neuroimaging visit in 1997–2000.^13,15,45^ Many of these participants (40 FASD, 26 HC) were also included in a separate analysis examining rates of cortical volumetric decline.^46^ Former study participants were located through the use of available contact information or other resources (e.g. Lexis/Nexis, TransUnion) to establish contact via letter, email, or phone and determine willingness to participate.

Twenty-six individuals in the FASD group were diagnosed with FAS as described by Streissguth *et al*.^45^ the criteria for which included evidence of dysmorphic features (e.g. flat philtrum, thin upper vermilion, flat midface, short palpebral fissures), growth deficiency of prenatal origin and evidence of a compromised CNS (e.g. microcephaly, delayed development, learning disabilities, etc.). Alcohol-affected individuals who did not meet full criteria for FAS were categorized as having fetal alcohol effects (FAE), a term which is no longer in use for individuals who may have lacked significant facial dysmorphology but still had a confirmed history of PAE and evidence of a compromised CNS. FASD subgroups were combined for primary analyses, as studies suggest that individuals with PAE are similar in terms of neuropsychological deficits and behaviour, independent of the presence or absence of facial dysmorphology.^47^ Individuals in the HC group were unexposed to alcohol prenatally. Lack of exposure in HC who participated in the original study was described in the original publications.^12,13^ Briefly, HC participants from the original study were excluded if they had difficulties with alcohol or drug use or if their biological mother had a history of alcohol or drug use or of binge drinking around the time of pregnancy. For the newly recruited HC, participants were asked if they had received a PAE-related diagnosis and whether, to their best of their knowledge, their mother drank alcohol, and if so, how frequently, during pregnancy. No other prenatal substance exposure questions (e.g. nicotine, cannabis) were asked of the newly recruited group. Those who were part of the original FASD Follow-up Study had been recruited from employees and their children at local healthcare facilities and schools to match the FASD group on age and ethnicity. For all participants, exclusion criteria included serious comorbid medical conditions (e.g. unstable hypertension or diabetes) and MRI contraindications (e.g. metal implants). Attention deficit-hyperactivity disorder and other developmental disorders were not exclusionary, but consistent with the original publication, other neurological problems were exclusionary. Lifetime psychiatric disorders were also not exclusionary and were not used to assess eligibility in the newly recruited HC participants. The study protocol was reviewed and approved by the University of Washington Institutional Review Board. All participants provided written informed consent. When necessary, consent was obtained from a legal guardian, and assent was obtained from the participant.

### Assessments

As part of the current study, participants completed measures from the NIH Toolbox Cognition Battery.^48^ Measures included the Dimensional Change Card Sort to assess set-shifting, the Flanker task to assess visuospatial inhibitory attention, List Sorting to examine working memory, Pattern Comparison to quantify processing speed, and Picture Sequence to measure episodic memory. These measures were combined to calculate a composite score of fluid intelligence, which reflects the ability to problem solve and adapt to novel situations. Importantly, fluid intelligence is believed to decline with age and may be sensitive to neurological integrity.^49^ Most participants also previously had completed the Wechsler Adult Intelligence Scale (WAIS-III) as part of their initial enrollment in the FASD Follow-up Study neuroimaging visit ∼25 years ago.

### Imaging procedures

#### Image acquisition

Data were collected using a 32-channel head coil on a 3 tesla Philips Ingenia CX scanner (Best, The Netherlands) housed at the University of Washington. Scan parameters for anatomical acquisitions were as follows: three-dimensional T_1_-weighted FFE (TR = 6.31 ms, TE = 2.9 ms, flip angle = 8°, 256 × 256 matrix, 1 × 1 × 1 mm^3^ resolution, FOV = 256 × 240); and a three-dimensional T2-weighted TSE (TR = 2500 ms, TE = 257.1 ms, flip angle = 90°, 256 × 256 matrix, 1 × 1 × 1 mm^3^ resolution, FOV = 256 × 240). The complete neuroimaging battery also included diffusion-weighted imaging and a resting state scan, which are not included in the current analysis.

#### Image preprocessing

Structural images were processed using the PreFreeSurfer, FreeSurfer and PostFreeSurfer minimal preprocessing pipelines from the Human Connectome Project.^50^ Supporting software included Workbench v1.4.2, HCPpipelines-4.0.0, and FreeSurfer v6. All images were inspected for data quality prior to processing. Post-processed images were inspected for artefacts and other errors. Three HC and eight FASD datasets were identified as having excessive motion or other image artefact and were thus excluded from all analyses, leaving a final sample of 28 HC and 48 FASD (24 with FAE, 24 with FAS). Volumetric data for the intracranial volume (ICV), corpus callosum, caudate, putamen, pallidum and cerebellum were estimated using FreeSurfer’s automatic segmentation.^51^ These regions were selected for consistency with our prior work that assessed age-related effects in largely the same group of participants who were scanned 25 years ago.^15^ As in our prior study using archival data,^15^ volumes from the left and right hemispheres for each ROI were summed to obtain bilateral estimates and to reduce the number of hypotheses tested.

### Data analysis

All statistical analyses were performed using R (https://www.r-project.org). Continuous independent variables were inspected for normality using skewness and kurtosis statistics and were mean-centered. Data were also examined for outliers using box plots; data were removed from analysis if their absolute value exceeded 1.5 times the interquartile range for the observed variable within each group. Data from two HC and three FASD group members were flagged as outliers in one or more regions of interest, and visual inspection confirmed these were due to questionable segmentations. Predictors of interest included exposure group, sex and age. Due to sample size, only linear effects of age were considered. Thus, the final model for each region included group, age, sex, group × age and group × sex. Where appropriate, *post hoc* pairwise comparisons of estimated marginal means were calculated with R’s *emmeans* package^52^ and False Discovery Rate corrected for multiple comparisons.^53^ The function *ggpredict* from the *ggeffects* R package^54^ was used to plot model predictions.

#### Clinical analysis

The HC and FASD groups were compared on age, body mass index (BMI), full-scale intelligence quotient (FSIQ) from the WAIS,^55^ and NIH Cognitive Toolbox fully corrected scores (T-scores) related to fluid intelligence using independent samples *t*-tests. Separate analyses examining group differences in completed education, sex, race and handedness were conducted using Chi-square tests, and level of education was examined using Fisher’s exact test.

#### Neuroimaging analysis

Data were analysed with and without correction for ICV. For analyses adjusting for ICV, the residualized method was used^15,56,57^ as follows: For each ROI, the HC group only was analysed using a regression model, where the ROI volume was the dependent variable and estimated ICV was the independent variable. The beta coefficient was subsequently used to generate residuals for each group that reflect the difference between each individual’s observed ROI volume and their predicted ROI volume. Consistent with current recommendations,^58^ we combined males and females when using the residual method to investigate sex differences, as differences in mean ICV between the sexes will impact normalization when sex is normalized separately.

#### Exploratory analysis

Given that the FASD sample was comprised of equal numbers of FAE and FAS, and that some studies suggest graded effects of volumetric reduction across the FASD spectrum.^46,59^ we also examined whether group, age, or sex differences may be specific to diagnosis. These analyses were exploratory due to the small sample size.

## Results

### Demographics

The HC and FASD groups did not differ on age, sex, or handedness (Table 1). The FASD group had significantly higher BMI relative to the HC group. Groups also significantly differed on completed education; while all HC completed one or more years of college, just over half of the individuals in the FASD group had one or more years of a college education. Most participants (22/28) from the HC group were employed full-time, whereas less than half (18/48) of those in the FASD group were employed full-time, *P* = 0.02. There were no significant differences in living arrangements, *P* = 0.77. One participant in the FASD group, and none in the HC group, stated that they had experienced trouble with the law. Groups did not differ on alcohol use, with AUDIT scores suggesting participants engaged in low-risk consumption patterns. Among individuals who participated in the original study,^13^ the HC group had higher FSIQ scores. Overall, the HC group also had a higher composite fluid intelligence score relative to the FASD group, and this was also reflected in subtest scores.

**Table 1:** Participant demographics and characteristics

Characteristic	HC (*N* = 28)	FASD (*N* = 48)	Statistics
Age (years)	43.2 (1.6)	40.8 (1.0)	*t*(45.2) = 1.27, *P* = 0.21, *d* = 0.324
Sex (M/F)	14/14	26/22	*χ* ^2^(1) = 0.01, *P* = 0.91, *ϕ* = 0.0001
BMI	26.9 (0.9)	30.0 (1.0)	*t*(71.3) = −2.21, *P* = 0.03, *d* = 0.481
FAS [*n* (%)]		24 (50%)	
Handedness [*n* (% right)]	25 (89%)	39 (83%)	*χ* ^2^(1) = 0.17, *P* = 0.68, *ϕ* = 0.002
Race			
American Indian/Alaskan Native	1	6	
Black/African American	2	2	
More than one race	1	10	
White	24	30	
Ethnicity			
Hispanic	1	2	
Non-Hispanic	27	43	
Unknown/Not reported	0	3	
Education			Fisher's exact test, *P* < 0.001
<High school	0	6	
High school graduate	0	11	
GED	0	5	
College+	28	26	
Employment			Fisher’s exact test, *P* = 0.02
Full-time	22	18	
Part-time, regular hours	2	5	
Part-time, variable hours	2	9	
Retired/Disability	1	9	
Student	0	1	
Unemployed	1	6	
Living arrangement^a^			Fisher’s exact test, *P* = 0.77
Alone	4	10	
With family	20	27	
With friends	0	1	
With roommate(s)	2	6	
No stable arrangement	0	1	
AUDIT	4.24 (2.88)	3.53 (4.70)	*t*(67.3) = 0.78, *P* = 0.44, *d* = 0.170
WAIS (baseline)			
FSIQ^b^	113.4 (2.7)	86.7 (2.3)	*t*(49.9) = 7.47, *P* < 0.001, *d* = 2.003
Verbal IQ**^b^**	112.0 (2.2)	85.1 (2.2)	t*(*53.0) = 8.71, *P* < 0.001, *d* = 2.287
Performance IQ**^b^**	112.5 (3.0)	91.2 (2.7)	*t*(50.8) = 5.24, *P* < 0.001, *d* = 1.397
NIH Toolbox^c^ (follow-up)			
Composite fluid score	59.1 (2.2)	40.9 (1.9)	*t*(50.5) = 6.22, *P* < 0.001, *d* = 1.611
Subtest scores			
Dimensional change card sort test	56.7 (2.4)	44.7 (1.7)	*t*(42.6) = 4.11, *P* < 0.001, *d* = 1.122
Flanker inhibitory control and attention test	48.3 (2.3)	37.3 (1.4)	*t*(38.2) = 4.10, *P* < 0.001, *d* = 1.156
Picture sequence memory test	60.5 (2.1)	49.9 (1.5)	*t*(43.3) = 4.18, *P* < 0.001, *d* = 1.135
List sorting working memory test	54.4 (1.8)	45.2 (2.0)	*t*(57.0) = 3.43, *P* = 0.001, *d* = 0.841
Pattern comparison processing speed test	61.2 (2.6)	42.4 (2.1)	*t*(48.4) = 5.62, *P* < 0.001, *d* = 1.476

Entries are of the form mean (standard error). Statistical comparisons were either by means of Welsh *t*-tests (effect sizes reported as Cohen’s *d*), *χ*^2^ test (*ϕ*) for equality of proportions or Fisher’s exact test.

AUDIT, Alcohol use disorders identification test; BMI, Body mass index; FASD, Fetal alcohol spectrum disorder; HC, Healthy control; WAIS, Wechsler Adult Intelligence Scale.

^a^2 HC, 3 FASD missing.

^b^4 HC, 15 FASD missing.

^c^5 HC, 11 FASD missing.

### Imaging analysis

Final samples for each analysis, including within group volume for each ROI and for ICV, are shown in Table 2. Linear regression results for each volume, using the raw values and after adjusting for ICV, are presented in Table 3.

**Table 2:** Average volumes in cubic millimetre for the healthy control (HC) and alcohol-exposed (FASD) groups

	Male				Female			
	HC		FASD		HC		FASD	
Region	*n*	Volume [*M* (SD)]	*n*	Volume [*M* (SD)]	*n*	Volume [*M* (SD)]	*n*	Volume [*M* (SD)]
Caudate	14	7118 (825)	26	6334 (996)	14	6789 (658)	22	5975 (980)
Putamen	13	10122 (916)	26	9096 (1323)	14	9157 (783)	21	8444 (1058)
Pallidum	13	3923 (180)	25	3526 (508)	14	3620 (397)	22	3227 (374)
Cerebellum	14	116493 (7913)	26	100138 (12132)	14	105816 (12478)	22	93892 (11984)
Corpus Callosum	14	3775 (299)	24	3621 (607)	14	3913 (489)	22	3145 (660)
ICV	14	1640422 (113156)	26	1485893 (164536)	14	1456948 (138015)	22	1337454 (137111)

Sample size *n* reflects the dataset used for the region’s analysis. Entries are of the form [mean (standard deviation)]. ICV, Intracranial vault.

**Table 3:** Regression results for the model examining age, sex, group, group × age and group × sex for both raw and ICV-controlled regional volumes

Region	Age	Sex	Group	Group × Age	Group × Sex
Caudate					
Raw volume	0.494 (0.036)	0.100 (0.050)	**<0.001 (0.180)**	0.807 (0.001)	0.947 (< 0.001)
*F*(1, 70)	0.47	2.78	**15.40**	0.06	0.00
ICV Residualized	0.250 (0.041)	0.715 (0.004)	**0.002 (0.124)**	0.703 (0.002)	0.818 (0.001)
*F*(1, 70)	1.35	0.13	**9.98**	0.14	0.04
Putamen					
Raw volume	0.589 (0.037)	**0.004 (0.131)**	**<0.001 (0.158)**	0.245 (0.024)	0.445 (0.009)
*F*(1, 68)	0.29	**8.77**	**12.75**	1.37	0.59
ICV Residualized	0.173 (0.047)	0.353 (0.014)	**0.046 (0.057)**	0.106 (0.042)	0.483 (0.007)
*F*(1, 68)	1.9	0.88	**4.14**	2.68	0.5
Pallidum					
Raw volume	0.536 (0.006)	**0.005 (0.126)**	**<0.001 (0.196)**	0.241 (0.021)	0.806 (0.001)
*F*(1, 68)	0.39	**8.49**	**16.55**	1.4	0.06
ICV Residualized	0.834 (0.002)	0.573 (0.006)	**0.008 (0.100)**	0.051 (0.050)	0.864 (< 0.001)
*F*(1, 68)	0.04	0.32	**7.58**	3.96	0.03
Cerebellum					
Raw volume	0.251 (< 0.001)	**0.011 (0.119)**	**<0.001 (0.275)**	0.105 (0.032)	0.594 (0.004)
*F*(1, 70)	1.34	**6.86**	**26.59**	2.69	0.29
ICV Residualized	0.532 (< 0.001)	0.749 (< 0.001)	**<0.001 (0.147)**	0.060 (0.045)	0.744 (0.002)
*F*(1, 70)	0.4	0.1	**12.09**	3.64	0.11
Corpus Callosum					
Raw volume	**0.026 (0.148)**	**0.016 (0.103)**	**<0.001 (0.202)**	0.221 (0.045)	**0.004 (0.113)**
*F*(1, 68)	**5.21**	**6.16**	**17.2**	1.53	**8.69**
ICV Residualized	**0.019 (0.146)**	**0.045 (0.075)**	**<0.001 (0.179)**	0.239 (0.043)	**0.004 (0.117)**
*F*(1, 68)	**5.73**	**4.16**	**14.85**	1.41	**9.01**

Values are presented as *P* ($\eta_{p}^{2}$). Significant (*P* < 0.05) values are shown in bold font. ICV, Intracranial volume.

#### Caudate

**Figure 1:**
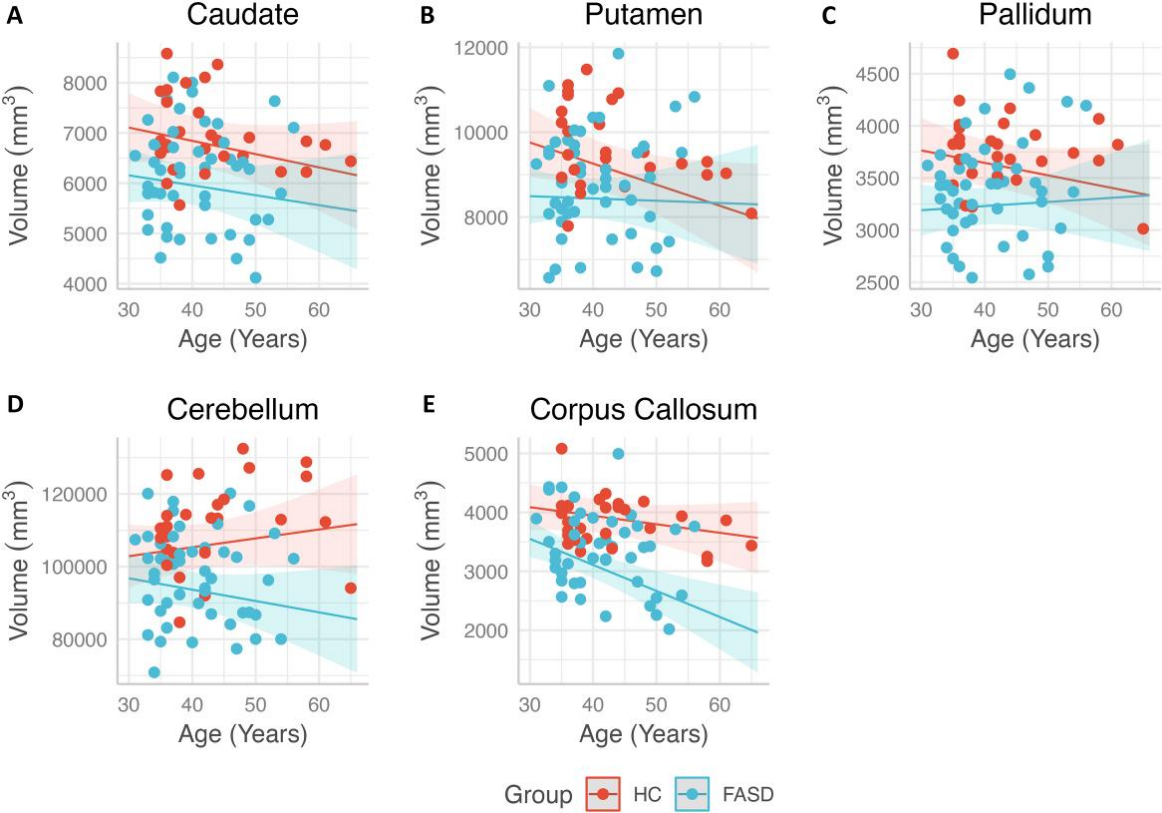
**ICV adjusted regional volumes across age for participants in the FASD and HC groups.** Within group trend lines represent linear regression model predictions for participant age (years) with shaded 95% confidence intervals for the residualized volumes (in cubic millimetre) of the regions of interest. While there were no statistically significant interactions of age with group within (**A**) the caudate and (**B**) the putamen (*P*s > 0.106), there was a trend for an interaction within the (**C**) pallidum (*F*(1.68) = 3.96, *P* = 0.051), and (**D**) cerebellum (*F*(1.70) = 3.64, *P* = 0.060). Similarly, there were no statistically significant interactions of age with group within the (**E**) corpus callosum (*P* = 0.239). FASD, Adults with fetal alcohol spectrum disorders; HC, Healthy comparison adults; ICV, Intracranial volume.

**Figure 2:**
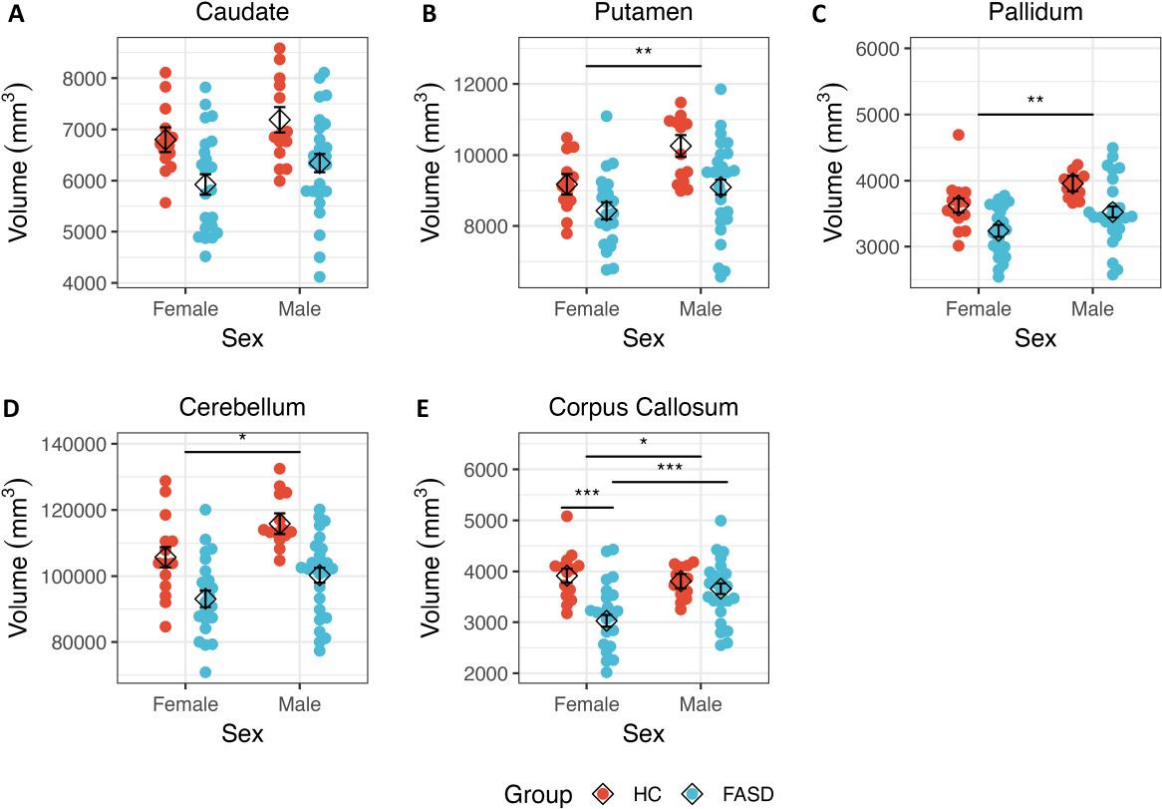
**Raw regional volumes plotted by sex for participants in the FASD and HC groups.** Beeswarm plots representing each individual’s raw regional volume (in cubic millimetre), stratified by sex and group, are shown, overlaid by the estimated marginal means (diamond shape) from the linear regression model. There was no main effect of sex in (**A**) the caudate (*P* > 0.05). However, there was a main effect of sex within the (**B**) putamen, (**C**) pallidum and (**D**) cerebellum. In addition to a main effect of sex, the (**E**) corpus callosum additionally showed an interaction of group with sex. Error bars represent standard error of the estimated marginal mean. *P < 0.05; **P < 0.01; ***P < 0.001. FASD, Adults with fetal alcohol spectrum disorders; HC, Healthy comparison adults.

**Figure 3:**
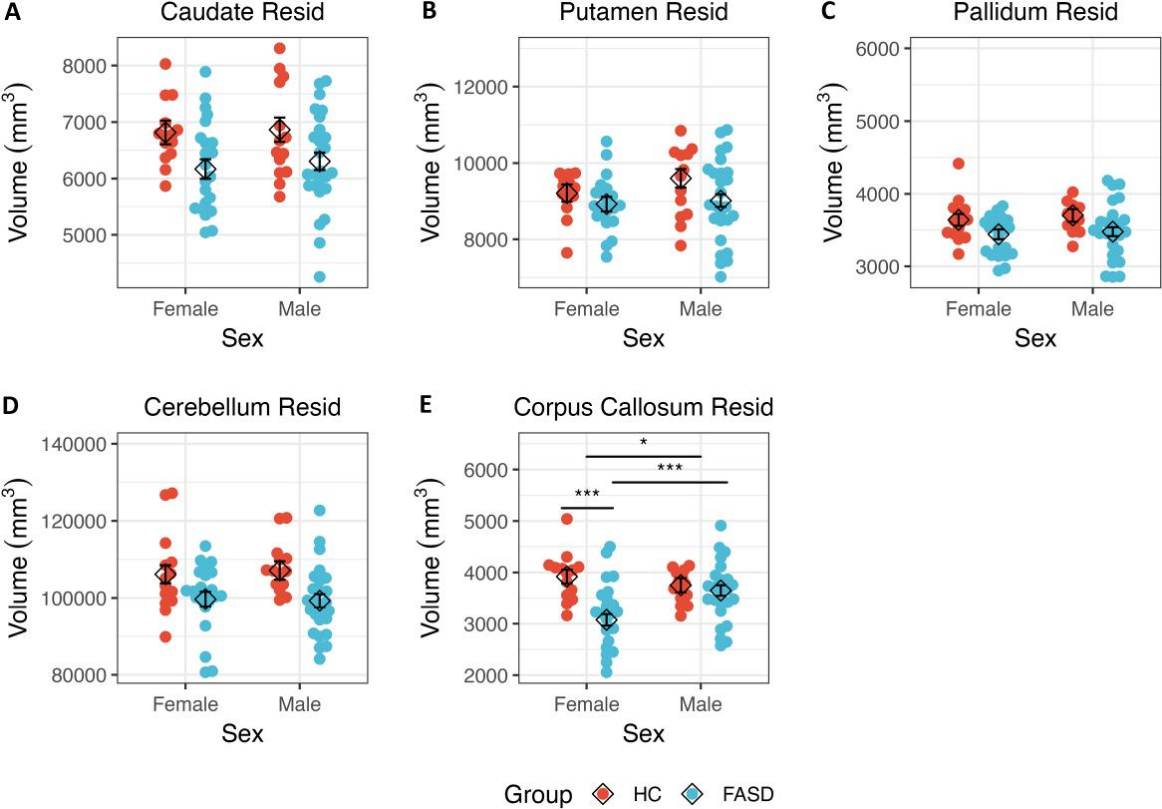
**ICV adjusted regional volumes plotted by sex for participants in the FASD and HC groups.** Beeswarm plots representing each individual’s ICV adjusted regional volume (in cubic millimetre), stratified by sex and group, are shown, and overlaid by the estimated marginal means (diamond shape) from the linear regression model. There was no main effect of sex within the (**A**) caudate, (**B**) putamen, (**C**) pallidum, or (**D**) cerebellum (*P*’s > 0.353). However, (**E**) the corpus callosum continued to show a main effect of sex as well as a group × sex interaction. Error bars represent standard error of the estimated marginal mean. *P < 0.05; ***P < 0.001. FASD, Adults with fetal alcohol spectrum disorders; HC, Healthy comparison adults; ICV, Intracranial volume; Resid, Residualized.

There was no significant interaction of group with age, nor was there a significant main effect for age (Fig. 1A). Similarly, there was no main effect of sex or significant interaction of group with sex (Fig. 2A). However, there was a significant main effect of group, whereby the HC group had a larger caudate volume relative to the FASD group. Significant findings were similar after adjusting the caudate volume for ICV (Fig. 3A).

#### Putamen

There was no main effect of age or significant interaction of group with age (Fig. 1B). Although there was no interaction of group with sex, there was a main effect of sex, whereby males had larger putamen volume relative to females (Fig. 2B). There was also a significant main effect of group, with the HC group having larger putamen volume relative to the FASD group. After adjusting putamen volume for ICV, only the main effect of group remained significant (Fig. 3B).

#### Pallidum

There was no main effect of age or interaction of group with age (Fig. 1C). There was a main effect of sex; male participants had larger volumes compared with female participants (Fig. 2C). However, this effect was no longer significant after adjusting the pallidum’s volume for ICV. No significant interaction of group with sex was detected. Finally, there was a main effect of group, such that the HC group had larger pallidal volumes relative to the FASD group, and this effect remained significant after adjusting for ICV (Fig. 3C).

#### Cerebellum

There was no main effect of age or significant interaction of age with group (Fig. 1D). There was a main effect of sex; as with other volumes, male participants had larger volumes relative to female participants (Fig. 2D), but this effect was not seen after controlling for ICV. There was no interaction of group with sex. However, there was a main effect of group; the HC group had larger volumes relative to the FASD group, and this effect held with adjustment for ICV (Fig. 3D).

#### Corpus callosum

There was a main effect of age, with older ages associated with smaller volumes (Fig. 1E), and this effect was consistent after adjusting for ICV. No interaction of group with age was detected. There was a main effect of sex (Fig. 2E), with males having larger volume relative to females, both with raw measures and after adjusting for ICV. There was a significant interaction of group with sex. *Post hoc* analyses suggested that females with FASD had smaller corpus callosum volumes relative to males with FASD [*t*(68) = 4.03, *P* < 0.001]. Moreover, females with FASD also had smaller corpus callosum volumes relative to HC females [*t*(68) = 4.98, *P* < 0.001]. This effect was also significant after controlling for ICV, with *post hoc* tests again showing that females with FASD had smaller corpus callosum volumes relative to both males with FASD [*t*(68) = 3.71, *P* < 0.001], and HC females [*t*(68) = 4.82, *P* < 0.001] (Fig. 3E). Finally, there was a main effect of group, whereby the HC group had larger volumes relative to the FASD group, and this effect held after adjusting for ICV.

#### Exploratory analyses

##### Demographics

Demographics and characteristics using three groups were similar to those seen when the FAS and FAE groups were combined (Supplementary Table 1). Both FAE and FAS were significantly different from the HC group on the FSIQ and other cognitive measures. BMI was also significantly different, with *post hoc* comparisons suggesting that the FAE group had higher BMI relative to the HC group [*t*(72) = 2.52, *P* = 0.04], but the FAS group was not significantly different from either the HC or FAE groups (*P*’s > 0.21).

##### Unpacking the main effect of group

When ROI models were repeated with three groups, all regions showed a main effect of group, consistent with our two-group analysis (Supplementary Table 2). *Post hoc* analyses suggested that, for all regions and both with and without accounting for ICV, the HC group had larger volumes relative to the FAS group [caudate_raw_: *t*(67) = 4.27, *P* < 0.001; caudate_resid_: *t*(67) = 3.33, *P* = 0.004; putamen_raw_: *t*(65) = 4.80, *P* < 0.001; putamen_resid_: *t*(65) = 2.99, *P* = 0.01]; pallidum_raw_: *t*(65) = 4.89, *P* < 0.001; pallidum_resid_: *t*(65) = 3.47, *P* = 0.003; cerebellum_raw_: *t*(67) = 6.78, *P* < 0.001; cerebellum_resid_: *t*(67) = 4.51, *P* < 0.001; corpus callosum_raw_: *t*(65) = 3.71, *P* = 0.001; corpus callosum_resid_: [*t*(65) = 3.71, *P* = 0.001]. The HC group also had larger volumes relative to the FAE group for the caudate [*t*(67) = 2.37, *P* = 0.03], pallidum [*t*(65) = 2.17, *P* = 0.03], cerebellum [*t*(67) = 3.06, *P* = 0.003] and corpus callosum [*t*(65) = 2.72, *P* = 0.01]. When accounting for ICV, only the corpus callosum continued to show that the HC group had a greater volume relative to the FAE group [*t*(65) = 2.72, *P* = 0.01]. Finally, the FAE group had larger volumes relative to the FAS group in the putamen [*t*(65) = 2.97, *P* = 0.006], pallidum [*t*(65) = 2.57, *P* = 0.02] and cerebellum [*t*(67) = 3.47, *P* = 0.001]. This finding held when ICV was taken into account within the putamen [*t*(65) = 2.28, *P* = 0.04] and cerebellum [*t*(67) = 2.52, *P* = 0.02].

##### Interactions with age

There was a significant interaction of age with group for both the pallidum and the cerebellum (*P*’s < 0.05). For the pallidum and only when accounting for ICV, *post hoc* analyses suggested the FAE group had a significantly different slope relative to the HC group (*P* = 0.04), with the FAE group showing increased volume with older age, and the HC group a decreased volume with older age (Supplementary Fig. 1). For the cerebellum for both the raw volume and when controlling for ICV, *post hoc* analyses suggested that slope differences were trending toward significance for HC relative to FAE (*P*’s < 0.09). *Post hoc* analyses did not suggest any significant slope differences between the FAE and FAS groups (*P*’s > 0.05).

##### Interactions with sex

Finally, only the corpus callosum showed a significant interaction of sex with group for both raw volume and with ICV correction (*P* = 0.009). *Post hoc* analyses suggested that, for raw volumes, males with FAS had larger volume relative to females with FAS [*t*(65) = 3.71, *P* = 0.002], and that HC females had larger volume relative to both females with FAE [*t*(65) = 3.15, *P* = 0.007] and females with FAS [*t*(65) = 5.14, *P* < 0.001]. These findings held when accounting for ICV: males with FAS had larger volume relative to females with FAS [*t*(65) = 3.38, *P* = 0.006], and female HC had larger volumes relative to females with FAE [*t*(65) = 3.11, *P* = 0.008] and to females with FAS [*t*(65) = 4.87, *P* < 0.001]. *Post hoc* analyses found no significant sex-related differences between the FAS and FAE groups.

## Discussion

To the best of our knowledge, this is one of the first studies to assess subcortical brain volumes in middle-aged adults with FASD. Our study found that relative to an age-matched HC group, adults with FASD had disproportionately smaller volumes of the caudate, putamen, pallidum, cerebellum and corpus callosum, which is consistent with previous research in younger cohorts from both our group and others.^15,28,60,61^ Contrary to our hypotheses and prior work in adolescents and young adults,^15^ however, we did not observe any interactions between age and group in our regions of interest analyses. These findings suggest that while volumetric reductions detected in early childhood persist into middle adulthood, there is no age-related decline between groups in middle age. Finally, although our findings largely support a lack of sexual dimorphism as observed in younger individuals with FASD in most regions, middle-aged females with FASD had smaller corpus callosum volumes than males with FASD, even after accounting for ICV. This unexpected finding suggests that PAE may have a greater impact on the corpus callosum in females than males in this age range, which in turn could have important implications for age-related cognitive decline for females with FASD.

Our findings of reduced subcortical volume, both with and without ICV adjustment, are consistent with the broader literature in children and adolescents with FASD. Multiple studies,^30,62-64^ including our prior work that scanned many of the same participants during adolescence,^15^ have reported reduced volumes for the caudate, pallidum and putamen, which may impact goal-directed learning and habitual behaviour.^65^ Others have associated reduced caudate volume with poorer neuropsychological performance and impaired inhibitory control in youth with heavy PAE.^66^ The head of the caudate is more connected with frontal regions,^67^ reflecting its role in cognitive function, whereas the tail of the caudate receives inputs primarily from temporal regions,^68,69^ potentially playing a greater role in visual discrimination and habit formation.^70-72^ The putamen is also engaged in cognitive function and is posited to be more associated with habitual behaviour, relative to the caudate and its role in more goal-directed behaviour.^65^ Although exploratory, we also found that the FAS group in particular had smaller volumes relative to the HC group within the caudate, putamen and pallidum, and similar findings have been reported in youth and adolescents.^15,64^ Others have reported that youth with PAE appear to have greater shape deformation throughout the caudate, and greater surface compression was associated with higher levels of alcohol exposure.^73^

Similarly, cerebellar volume in individuals with FASD was reduced relative to the age-matched HC group. Reduced cerebellar volume is also a common finding in children, adolescents and young adults with PAE,^60-62,64^ including when our current cohort was studied in adolescence.^15^ Others have reported that lower cerebellar volumes were associated with higher levels of PAE or dysmorphia,^11,14,15^ which we also found in our exploratory analyses.

We also demonstrated that older individuals with FASD continue to show reduced volumes of the corpus callosum relative to age-matched HC adults. The corpus callosum was among the first brain regions noted to be affected by PAE in early autopsy studies and subsequently confirmed using neuroimaging.^74-76^ Corpus callosum morphology in FASD was studied in detail by Bookstein *et al*.,^12,13,77^ and it was found that callosal shape was highly variable among adolescents and young adults with FASD and predicted neuropsychological deficits. In a secondary analysis of the same dataset, and which overlaps our current group when they were younger, our group reported that the volume of the corpus callosum was smaller in adolescents and young adults with FASD relative to HC.^15^ Taken together, these findings support our hypothesis that reduced volumes persist into middle age in individuals with FASD.

Our hypothesis of a group-by-age interaction within our regions of interest was not supported. These findings stand in contrast with our prior cross-sectional analyses that included largely the same participants during adolescence and young adulthood.^15^ Our earlier work suggested a quadratic effect with age in the FASD group, whereby corpus callosum volume increases peaked in early adulthood before beginning to show volumetric decreases. This difference in findings may be partly due to the current analysis involving a smaller sample size than the original study, which had included 107 individuals with FASD and 56 HC individuals, or it may suggest less age-related variability between groups in adulthood. Studies of regional volume across the lifespan in healthy individuals generally show reductions in cortical grey matter.^78,79^ While subcortical regions also decline after reaching their childhood peak,^80^ the rate of decline, while overall slower than the cortex, show regional variability.^81^ For example, cross-sectional studies suggest that the putamen’s volume reduces by up to 20% over the lifespan, whereas the caudate’s volumetric decline is more modest.^22,82-84^ The cerebellum also shows volumetric decline during normal aging.^26,85-87^ Although our exploratory analyses did suggest there may be age-related group differences within the cerebellum and pallidum, mainly with the FAE group, this should be interpreted with caution given the small sample size.

Few studies have examined sex-related differences in individuals with PAE, in part due to relatively small sample sizes. However, those studies that have examined sex-related effects have generally noted a lack of sexual dimorphism in individuals with FASD. In a relatively large cohort (74 HC, 70 with FASD) that included participants ranging from childhood to early adulthood, Treit *et al*.^34^ reported a significant interaction of sex with group. While HC participants had larger thalamus, putamen and caudate volumes relative to participants with FASD, these differences were more pronounced in males than in females. Others have reported a lack of sexual dimorphism in the pituitary gland volume of adolescents with FASD.^88^ Our prior cross-sectional study of the same participants during adolescence and young adulthood^15^ also did not show sex-related differences in adolescents and young adults. However, some studies have reported differences in white matter microstructure. For example, male children and adolescents with PAE had higher fractional anisotropy (FA) in subregions of the corpus callosum relative to females with PAE, suggesting that males with PAE may have better white matter integrity than females with PAE.^89^ Others have reported lower FA in the genu of the corpus callosum in female children with PAE relative to HC females.^90^ In comparison, our findings in older adults suggest that females with FASD had disproportionately lower corpus callosum volume relative to HC females, even after controlling for ICV. There were no significant sex-related differences in other volumes examined in this study. Structural variability of the brain across the lifespan has been reported for healthy individuals, and males appear to exhibit greater variance relative to females in most regions.^91,92^ For example, corpus callosum volume increases for both males and females until adulthood, with females exhibiting a slightly longer trajectory of development into their 20s before exhibiting evidence of volumetric decrease, whereas volume in males tends to remain stable before beginning to decline in their 40s.^81^ Others suggest there is no sexual dimorphism of any structure in healthy adults; rather, any perceived differences by sex are outweighed by other factors that may contribute to variability.^93,94^ In support of this, we did not detect sex-related differences between males and females in the HC group in *post hoc* analyses. Instead, sex-related differences were associated with PAE and suggested that in middle adulthood, females with FASD were particularly affected, whether in comparison to males with FASD or HC females. Aging is a complex biological process, and there is increasing evidence linking reproductive health with future dementia risk.^95-97^ In healthy adults, lower white matter integrity within the corpus callosum has previously been associated with poorer cognitive performance, as well as age-related decline.^98-100^ White matter integrity within the body of the corpus callosum has also been associated with motor learning task performance in healthy adults,^101^ with more recent work suggesting that sex may impact corpus callosum neuroplasticity during motor learning.^102^ Our finding of disproportionately lower corpus callosum volume in females with FASD relative to HC females could be indicative of greater age-related decline, placing females with FASD at higher risk for future cognitive impairment as they age. Longitudinal neuroimaging studies would help address this question.

Our study had several limitations. The sample size was small, and more studies are needed with larger samples and multiple time points to determine whether there are nonlinear effects due to age. Although the majority of participants were also part of the original imaging study 25 years ago, longitudinal analyses were not pursued due to substantial differences in scanner and acquisition parameters. There has been relatively little work on longitudinal harmonization for neuroimaging. Recent research in this area suggests scan protocols, including hardware, software and sequence parameters should be carefully and fully documented, and that sample sizes be sufficiently balanced.^103^ Moreover, 12% of the current sample did not participate in the original neuroimaging study; this, along with the lack of a third imaging time point to calculate trajectory, informed our decision to pursue a cross-sectional subcortical analysis. However, other recent work in an overlapping study sample using a data harmonization method to assess cortical volume across two time points also reported overall reduced cortical volume for the FASD group relative to the HC group.^46^ Together, these results argue for additional time points, and future longitudinal work is planned to address this critical gap. Alternatively, other approaches, such as the brain age gap,^104^ may be helpful in assessing age-related differences. BMI is also a potential confound, as greater BMI has also been associated with lower subcortical volume.^105^ As BMI was confounded with group, it was not included in our models. Future studies should consider inclusion of individuals with both normal weight and overweight/obesity to address this issue. Participants were in middle adulthood, and the mean age was in the early 40’s. Large-scale studies in healthy populations suggest that subcortical regions experience a modest volumetric decline with age that accelerates in the sixth decade.^80^ Finally, it is possible the sample was biased toward individuals with less pronounced effects of PAE, as those with more pronounced effects may have experienced disability-related barriers to study participation. However, this work was also constrained by the impact of the COVID-19 pandemic. It is therefore also possible that higher functioning individuals with FASD may have been less able to participate due to other constraints on their availability, whereas lower functioning individuals may have had greater caregiver support that supported study participation.^43^ Both possibilities impact study generalizability.

## Conclusion

Individuals with FASD show reduced subcortical volumes in midlife, suggesting that PAE may confer lifelong consequences to brain structure. In addition to ongoing behavioural and cognitive deficits, altered neurological structure may have implications for future health as these individuals age. It will be important to consider whether early interventions may lessen the overall impact of PAE across the lifespan.

## Supplementary Material

fcae273_Supplementary_Data

## Data Availability

The data that support the findings of this study are available from the corresponding author, upon reasonable request. The preprocessing code used to support this study can be found at https://github.com/Washington-University/HCPpipelines.
