# Supplementary material for: Subcortical volume in middle-aged adults with fetal alcohol spectrum disorders
Source: Brain Commun. 2024 Sep 3;6(5):fcae273. doi: 10.1093/braincomms/fcae273 (PMC11369821; doi:10.1093/braincomms/fcae273)

**Subcortical volume in middle-aged adults with fetal alcohol spectrum disorders**

Amanda Bischoff-Grethe^1^, Susan A. Stoner^2^, Edward P. Riley^3^, Eileen M. Moore^3^

^1^Department of Psychiatry, University of California, San Diego, La Jolla, California, 92093, USA

^2^Fetal Alcohol and Drug Unit, Department of Psychiatry and Behavioral Sciences, University of Washington School of Medicine, Seattle, Washington, 98105 USA

^3^Center for Behavioral Teratology, Department of Psychology, San Diego State University, San Diego, California, 92120, USA

Correspondence to: Amanda Bischoff-Grethe, Ph.D.

Department of Psychiatry

University of California, San Diego

9500 Gilman Drive, MC 0738

La Jolla, CA 92093-0738

USA

Tel: 858-246-0604

agrethe@health.ucsd.edu

Supplemental Material

Supplemental Tables: 2

Supplemental Figures: 1

**Supplemental Table 1. Participant demographics and characteristics by diagnosis.**

|  | **HC (n = 28)** | **FAE (n = 24)** | **FAS (n = 24)** | **Statistics** | **Post Hoc Comparison** |
| --- | --- | --- | --- | --- | --- |
| Age (years) | 43.2 (1.6) | 39.9 (1.4) | 41.6 (1.3) | F(2,73) = 1.24, P = 0.295, r^2^ = 0.033 |  |
| Sex (M/F) | 14/14 | 13/11 | 13/11 | χ^2^(2) = 0.123, P = 0.94, Cramer's V = 0.04 |  |
| BMI | 26.9 (0.9) | 31.4 (1.5) | 28.6 (1.4) | F(2,72) = 3.19, P = 0.047, r^2^ = 0.081 | FAE > HC* |
| Handedness [n (% right)] | 25 (89%) | 22 (92%) | 18 (75%) | χ^2^(2) = 3.20, P = 0.20, Cramer's V = 0.21 |  |
| **Race** |  |  |  |  |  |
| American Indian/Alaskan Native | 1 | 2 | 4 |  |  |
| Black/African American | 2 | 0 | 2 |  |  |
| More Than One Race | 1 | 5 | 5 |  |  |
| White | 24 | 17 | 13 |  |  |
| Ethnicity |  |  |  |  |  |
| Hispanic | 1 | 1 | 1 |  |  |
| Non-Hispanic | 27 | 21 | 22 |  |  |
| Unknown/Not Reported | 0 | 2 | 1 |  |  |
| **Education** |  |  |  | Fisher's Exact Test, P < 0.001 |  |
| < High School | 0 | 0 | 6 |  |  |
| High School Graduate | 0 | 2 | 3 |  |  |
| GED | 0 | 6 | 5 |  |  |
| College+ | 28 | 16 | 10 |  |  |
| **WAIS^a^ (baseline)** |  |  |  |  |  |
| FSIQ | 113.4 (2.7) | 91.3 (3.5) | 83.8 (3.0) | F(2,54) = 29.99, P < 0.001, r^2^ = 0.526 | HC > FAE, FAS*** |
| Verbal IQ | 112.0 (2.2) | 89.3 (3.5) | 82.3 (2.6) | F(2,54) = 39.01, P < 0.001, r^2^ = 0.591 | HC > FAE, FAS*** |
| Performance IQ | 112.5 (3.0) | 94.8 (4.4) | 88.9 (3.4) | F(2,54) = 14.18, P < 0.001, r^2^ = 0.344 | HC > FAE, FAS*** |
| **NIH Toolbox ^b^ (follow-up)** |  |  |  |  |  |
| Composite Fluid Score | 59.1 (2.2) | 41.3 (2.7) | 40.5 (2.9) | F(2,57) = 18.12, P < 0.001, r^2^ = 0.389 | HC > FAE, FAS*** |
| Subtest Scores |  |  |  |  |  |
| Dimensional Change Card Sort Test | 56.7 (2.4) | 44.9 (2.7) | 44.5 (2.1) | F(2,57) = 8.78, P < 0.001, r^2^ = 0.235 | HC > FAE, FAS** |
| Flanker Inhibitory Control and Attention Test | 48.3 (2.3) | 36.9 (2.0) | 37.7 (2.0) | F(2,57) = 9.37, P < 0.001, r^2^ = 0.247 | HC > FAE, FAS** |
| Picture Sequence Memory Test | 60.5 (2.1) | 52.0 (1.8) | 48.0 (2.3) | F(2,57) = 10.13, P < 0.001, r^2^ = 0.262 | HC > FAE, FAS*** |
| List Sorting Working Memory Test | 54.4 (1.8) | 46.1 (2.1) | 44.4 (3.4) | F(2,57) = 5.05, P = 0.01, r^2^ = 0.150 | HC > FAE, FAS* |
| Pattern Comparison Processing Speed Test | 61.2 (2.6) | 41.8 (3.0) | 42.9 (3.1) | F(2,57) = 15.23, P < 0.001, r^2^ = 0.348 | HC > FAE, FAS*** |
| Entries are of the form mean (standard error). Statistical comparisons were either by means of simple linear regression (lm function in R), χ^2^ test (Cramer's V) for equality of proportions, or Fisher’s Exact Test. Where appropriate, post hoc pairwise comparisons of estimated marginal means were determined with R's emmeans package and false discovery rate corrected for multiple comparisons. BMI: body mass index; FAE: fetal alcohol effects; FAS: fetal alcohol syndrome; HC: healthy control; WAIS: Wechsler Adult Intelligence Scale.  ^a^4 HC, 11 FAE, 4 FAS missing  ^b^5 HC, 6 FAE, 5 FAS missing  *P < 0.05, **P < 0.01, ***P < 0.001 | | | | | |

**Supplemental Table 2. Regression results for the model examining Age, Sex, Group (HC, FAE, FAS), Group × Age, and Group × Sex for both raw and ICV-controlled regional volumes.**

| **Region** | **Age** | **Sex** | **Group** | **Group × Age** | **Group × Sex** |
| --- | --- | --- | --- | --- | --- |
| **Caudate** |  |  |  |  |  |
| Raw volume | 0.49 (0.028) | 0.10 (0.050) | **< 0.001 (0.212)** | 0.80 (0.015) | 0.29 (0.036) |
|  | F(1,67) = 0.49 | F(1,67) = 2.87 | **F(2,67) = 9.02** | F(2,67) = 0.22 | F(2,67) = 1.26 |
| ICV Residualized | 0.25 (0.035) | 0.71 (0.003) | **0.006 (0.142)** | 0.57 (0.026) | 0.36 (0.030) |
|  | F(1,67) = 1.37 | F(1,67) = 0.14 | **F(2,67) = 5.34** | F(2,67) = 0.58 | F(2,67) = 1.04 |
| **Putamen** |  |  |  |  |  |
| Raw volume | 0.57 (0.029) | **0.003 (0.148)** | **< 0.001 (0.252)** | 0.30 (0.042) | 0.42 (0.027) |
|  | F(1,65) = 0.32 | **F(1,65) = 9.67** | **F(2,65) = 10.93** | F(2,65) = 1.24 | F(2,65) = 0.89 |
| ICV Residualized | 0.17 (0.040) | 0.35 (0.015) | **0.02 (0.116)** | 0.19 (0.058) | 0.64 (0.014) |
|  | F(1,65) = 1.97 | F(1,65) = 0.91 | **F(2,65) = 4.26** | F(2,65) = 1.72 | F(2,65) = 0.45 |
| **Pallidum** |  |  |  |  |  |
| Raw volume | 0.52 (0.002) | **0.004 (0.128)** | **< 0.001 (0.259)** | 0.29 (0.045) | 0.58 (0.017) |
|  | F(1,65) = 0.41 | **F(1,65) = 9.02** | **F(2,65) = 11.34** | F(2,65) = 1.27 | F(2,65) = .56 |
| ICV Residualized | 0.83 (0.00) | 0.57 (0.004) | **0.007 (0.143)** | **0.049 (0.090)** | 0.92 (0.003) |
|  | F(1,65) = 0.05 | F(1,65) = 0.332 | **F(2,65) = 5.41** | **F(2,65) = 3.17** | F(2,65) = 0.085 |
| **Cerebellum** |  |  |  |  |  |
| Raw volume | 0.20 (0.002) | **0.005 (0.153)** | **< 0.001 (0.414)** | **0.027 (0.072)^a^** | 0.32 (0.033) |
|  | F(1,67) = 1.68 | **F(1,67) = 8.57** | **F(2,67) = 23.72** | **F(2,67) = 3.81** | F(2,67) = 1.16 |
| ICV Residualized | 0.51 (0.003) | 0.734 (0.00) | **< 0.001 (0.247)** | **0.032 (0.078)^a^** | 0.689 (0.011) |
|  | F(1,67) = 0.447 | F(1,67) = 0.116 | **F(2,67) = 10.98** | **F(2,67) = 3.62** | F(2,67) = 0.37 |
| **Corpus Callosum** |  |  |  |  |  |
| Raw volume | **0.026 (0.135)** | **0.016 (0.101)** | **< 0.001 (0.226)** | 0.56 (0.037) | **0.009 (0.135)** |
|  | **F(1,65) = 5.20** | **F(1,65) = 6.15** | **F(2,65) = 9.47** | F(2,65) = 0.59 | **F(2,65) = 5.08** |
| ICV Residualized | **0.020 (0.134)** | **0.047 (.072)** | **< 0.001 (0.199)** | 0.581 (0.036) | **0.009 (0.134)** |
|  | **F(1,65) = 5.67** | **F(1,65) = 4.11** | **F(2,65) = 8.05** | F(2,65) = 0.55 | **F(2,65) = 5.04** |
| \| Values are presented as *P* ($\eta_{p}^{2}$). Significant (*P* < 0.05) values are shown in bold font. ICV: intracranial volume ^a^Group x Age interaction was significant in the three-group analysis, but not for the two-group analysis. \| \| --- \| | | | | | |

**Supplemental Figure 1. ICV adjusted regional volumes across age for participants in the FAE, FAS, and HC groups.** Within group trend lines represent linear regression model predictions for participant age (years) with shaded 95% confidence intervals for the residualized volumes (mm*3*) of the regions of interest. There were no statistically significant interactions of age with group within the (A) caudate or (B) putamen (*P*s > 0.19). However, there was a significant interaction within the (C) pallidum (*F*(2,65) = 3.17, *P* = 0.049) and (D) cerebellum (F(2,67) = 3.62, *P* = 0.032). . Post hoc analyses using R’s *emtrends* suggested the FAE group had a significantly different slope relative to the HC group (*P* = 0.04) in the pallidum and a nonsignificant trend for slope differences for HC relative to FAE (*P*’s < 0.09). No statistically significant interaction of age with group was detected for the (E) corpus callosum (XS). Abbreviations: FAE: adults with fetal alcohol effects; FAS: adults with fetal alcohol syndrome; HC: healthy comparison adults; ICV: intracranial volume; Resid: residualized.


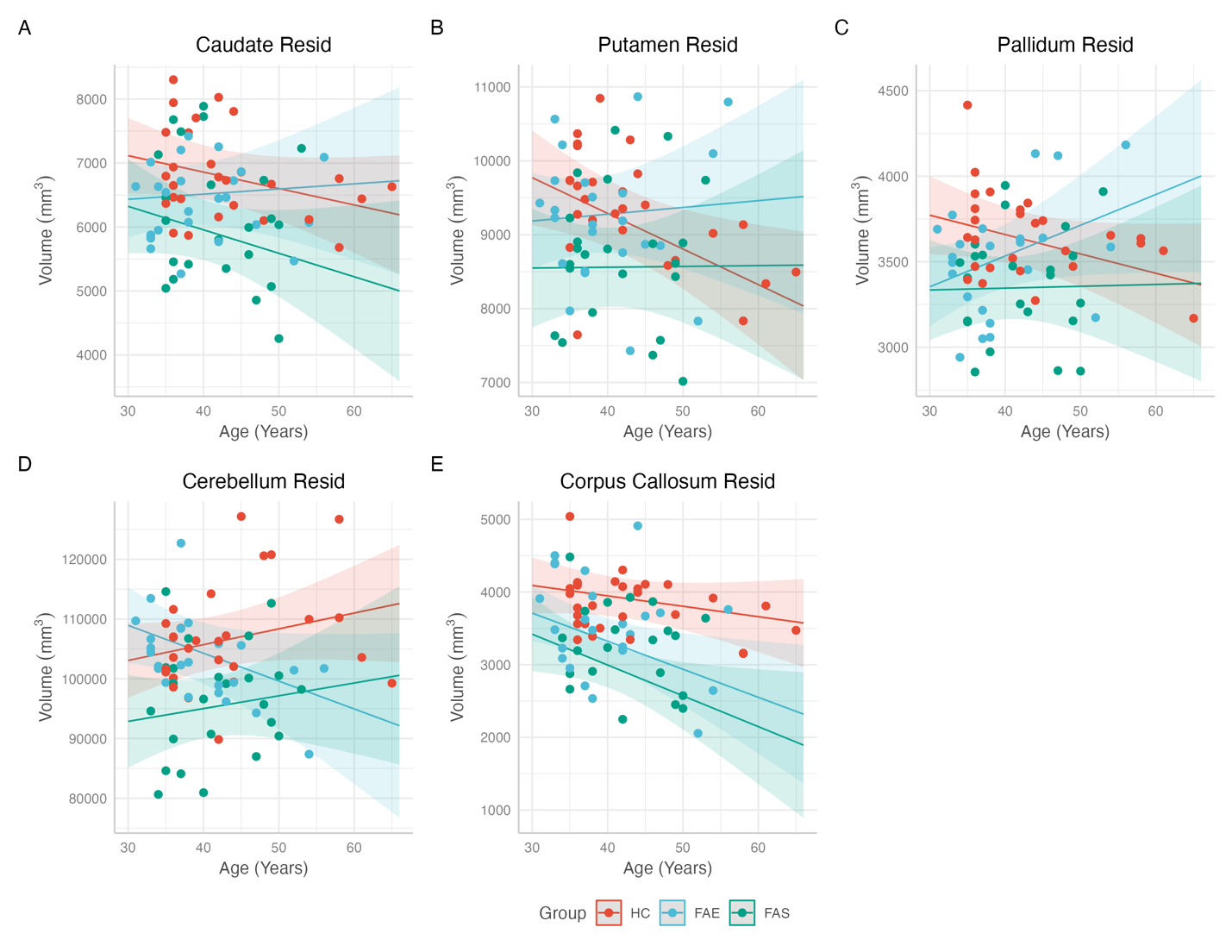

Supplement: fcae273_Supplementary_Data [file fcae273_supplementary_data.docx]
